# Activation of fibroblast-like synoviocytes derived from rheumatoid arthritis via lysophosphatidic acid–lysophosphatidic acid receptor 1 cascade

**DOI:** 10.1186/s13075-014-0461-9

**Published:** 2014-10-02

**Authors:** Yoshishige Miyabe, Chie Miyabe, Yoshiko Iwai, Waka Yokoyama, Chiyoko Sekine, Kazutaka Sugimoto, Masayoshi Harigai, Masayuki Miyasaka, Nobuyuki Miyasaka, Toshihiro Nanki

**Affiliations:** Department of Medicine and Rheumatology, Graduate School of Medical and Dental Sciences, Tokyo Medical and Dental University, 1-5-45, Yushima, Bunkyo-ku, Tokyo 113-8519 Japan; Department of Dermatology, Tokyo Medical University, 6-7-1, Nishi-Shinjuku, Shinjuku-ku, Tokyo 160-0023 Japan; Department of Molecular Biology, School of Medicine, University of Occupational and Environmental Health, 1-1 Iseigaoka, Yahatanishi-ku, Kitakyushu, Fukuoka 807-8555 Japan; Department of Clinical Research Medicine, Teikyo University, 2-11-1, Kaga, Itabashi-ku, Tokyo 173-8605 Japan; Sonoda Joint Replacement and Sports Medical Center, 1-21-10, Hogima, Adachi-ku, Tokyo 121-0064 Japan; Department of Pharmacovigilance, Graduate School of Medical and Dental Sciences, Tokyo Medical and Dental University, 1-5-45, Yushima, Bunkyo-ku, Tokyo 113-8519 Japan; Interdisciplinary Program for Biomedical Sciences, Osaka University, 2-2, Yamada-oka, Suita, Osaka 565-0871 Japan

## Abstract

**Introduction:**

Lysophosphatidic acid (LPA) is a bioactive lipid that binds to G protein–coupled receptors (LPA_1–6_). Recently, we reported that abrogation of LPA receptor 1 (LPA_1_) ameliorated murine collagen-induced arthritis, probably via inhibition of inflammatory cell migration, Th17 differentiation and osteoclastogenesis. In this study, we examined the importance of the LPA–LPA_1_ axis in cell proliferation, cytokine/chemokine production and lymphocyte transmigration in fibroblast-like synoviocytes (FLSs) obtained from the synovial tissues of rheumatoid arthritis (RA) patients.

**Methods:**

FLSs were prepared from synovial tissues of RA patients. Expression of LPA_1–6_ was examined by quantitative real-time RT-PCR. Cell surface LPA_1_ expression was analyzed by flow cytometry. Cell proliferation was analyzed using a cell-counting kit. Production of interleukin 6 (IL-6), vascular endothelial growth factor (VEGF), chemokine (C-C motif) ligand 2 (CCL2), metalloproteinase 3 (MMP-3) and chemokine (C-X-C motif) ligand 12 (CXCL12) was measured by enzyme-linked immunosorbent assay. Pseudoemperipolesis was evaluated using a coculture of RA FLSs and T or B cells. Cell motility was examined by scrape motility assay. Expression of adhesion molecules was determined by flow cytometry.

**Results:**

The expression of LPA_1_ mRNA and cell surface LPA_1_ was higher in RA FLSs than in FLSs from osteoarthritis tissue. Stimulation with LPA enhanced the proliferation of RA FLSs and the production of IL-6, VEGF, CCL2 and MMP-3 by FLSs, which were suppressed by an LPA_1_ inhibitor (LA-01). Ki16425, another LPA_1_ antagonist, also suppressed IL-6 production by LPA-stimulated RA FLSs. However, the production of CXCL12 was not altered by stimulation with LPA. LPA induced the pseudoemperipolesis of T and B cells cocultured with RA FLSs, which was suppressed by LPA_1_ inhibition. In addition, LPA enhanced the migration of RA FLSs and expression of vascular cell adhesion molecule and intercellular adhesion molecule on RA FLSs, which were also inhibited by an LPA_1_ antagonist.

**Conclusions:**

Collectively, these results indicate that LPA–LPA_1_ signaling contributes to the activation of RA FLSs.

## Introduction

Rheumatoid arthritis (RA) is a chronic inflammatory disease characterized by synovial hyperplasia with proliferation of fibroblast-like synoviocytes (FLSs), angiogenesis, infiltration of inflammatory cells such as lymphocytes and macrophages, and bone destruction of multiple joints [1]. FLSs are especially responsible for inflammation through cytokine and chemokine production and are also key cells of the invasive synovium, suggesting that they play a major role in the initiation and perpetuation of the destruction of inflamed joints [2].

Lysophosphatidic acid (LPA) is a bioactive lipid that binds to its specific cell surface G protein–coupled receptors (LPA_1–6_). LPA is generated via the hydrolysis of lysophosphatidylcholine by a secretory protein, autotaxin (ATX), which exhibits lysophospholipase D activity [3]. ATX was shown to be highly expressed in tumor cells, including neuroblastoma, breast cancer and renal cell carcinoma [4-6]. Moreover, LPA was reported to induce the production of interleukin 8 (IL-8) and vascular endothelial growth factor (VEGF) by cancer cells, angiogenesis and cancer growth [7-11].

It has previously been shown that expression of ATX by FLSs in the RA synovium and concentration of ATX in the RA synovial fluid are increased [12]. In addition, LPA_1–3_ mRNA has been reported to be expressed in RA FLSs, and incubation with LPA induced cell motility and cytokine expression by the FLSs, indicating that LPA may contribute to the pathogenesis of RA by stimulation of FLSs [13,14]. We recently demonstrated that treatment with an LPA receptor 1 (LPA_1_) antagonist, LA-01, ameliorated murine collagen-induced arthritis, probably via inhibition of inflammatory cell migration, Th17 differentiation and osteoclastogenesis [15].

In this study, we extensively analyzed the stimulatory effects of LPA for RA FLSs, as well as the effects of an LPA_1_ antagonist, LA-01, against this stimulation.

## Methods

### Specimens

Synovial tissues were obtained from RA patients (*n* = 10) who fulfilled American College of Rheumatology criteria [16] and from patients with osteoarthritis (OA) (*n* = 5). RA patients were a median (range) of 67 years old (45 to 80), and had a disease duration of 14 years (2 to 30) and C-reactive protein level of 0.68 mg/dl (0.0 to 2.85). Seven patients (70%) were positive for rheumatoid factor, and seven (70%) were positive for anticitrullinated protein antibodies. All patients provided informed consent. The experimental protocol was approved by the ethics committee of the Tokyo Medical and Dental University.

### Fibroblast-like synoviocytes

Synovial tissues from RA patients were minced and incubated with 0.5 mg/ml collagenase (Sigma-Aldrich, St Louis, MO, USA) for 1 hour at 37°C, then passed through a metal screen to obtain single-cell suspensions. Harvested cells were plated in cell culture plates and incubated with Dulbecco’s modified Eagle’s medium (DMEM) (Sigma-Aldrich) supplemented with 10% fetal calf serum (FCS) (Sigma-Aldrich). Adherent cells were maintained in the medium as FLSs and were used after five passages in the following experiments [17].

### RT-PCR

Total RNA was prepared from the FLSs of RA tissue (*n* = 10) and OA synovial tissue (*n* = 5), and first-strand cDNA was synthesized. Quantitative real-time RT-PCR was performed as described previously [18]. cDNA was amplified with primers for LPA_1_ (sense, 5′-ACC CAA TAC TCG GAG ACT GAC TGT-3′; antisense, 5′-CGT CAG GCT GGT GTC AAT GA-3′), LPA_2_ (sense, 5′-TCA TCA TGG GCC AGT GCT ACT-3′; antisense, 5′-GTG GGA GCT GAG CTC TTT GC-3′), LPA_3_ (sense, 5′-CTT GAC TGC TTC CCT CAC CAA-3′; antisense, 5′-CGC ATC CTC ATG ATT GAC ATG-3′), LPA_4_ (sense, 5′-TCC TCA GTG GCG GTA TTT CAG-3; antisense, 5′-AAG CAG GTG GTG GTT GCA TT-3′), LPA_5_ (sense, 5′-GGT GGT GAG CGT GTA CAT GTG T-3′; antisense, 5′-AGT GGT GCA GTG CGT AG TAG GA-3′), LPA_6_ (sense, 5′-AGA ACC AAA AGA AAT GCA AAG ATT G-3′; antisense, 5′-ACG GCG GGT GCA CTT C-3′) and 18S rRNA (sense, 5′-AAC CAG ACA AAT CGC TCC AC-3′; antisense, 5′-ACT CAA CAC GGG AAA CCT CA-3′). 18S rRNA was used as an internal control to standardize the amount of sample mRNA, and the relative expression of real-time PCR products was determined.

### Cell surface expression of lysophosphatidic acid receptor 1 on fibroblast-like synoviocytes

FLSs were stained with anti-LPA_1_ monoclonal antibody (mAb) (1G6; LSBio, Seattle, WA, USA) as a first antibody, and phycoerythrin-conjugated anti-mouse immunoglobulin G (IgG) antibody (BioLegend, San Diego, CA, USA) as a second antibody. Mouse IgG2b (BioLegend) was used as an isotype control. Cells were then analyzed by flow cytometry (FACSCalibur; BD Biosciences, San Jose, CA, USA).

### Proliferation assay

FLSs were plated at a density of 2 × 10^3^ cells/well in 96-well flat-bottom plates. Cells were incubated with a selective LPA_1_ antagonist (LA-01 (0, 1 or 10 nM); provided by Ono Pharmaceutical, Osaka, Japan) [15,19] for 30 minutes and then stimulated with LPA (Cayman Chemical, Ann Arbor, MI, USA) (0, 1 or 10 μM) in FCS-free DMEM at 37°C for 72 hours. The proliferation of FLSs was measured by using a cell-counting kit with WST-8 (2-(2-methoxy-4-nitrophenyl)-3-(4-nitrophenyl)-5-(2,4-disulfophenyl)-2H-tetrazolium, monosodium salt; Dojindo, Kumamoto, Japan) according to the manufacturer’s protocol. LPA_1_, LPA_2_ and LPA_3_ share 50% to 57% amino acid identity in humans and comprise the endothelial cell differentiation gene (Edg) family of LPA receptors [20]. The half-maximal inhibitory concentration (IC50) of LA-01 was 0.086, 2.8 and 0.90 μmol/L for LPA_1_, LPA_2_ and LPA_3_, respectively, which was determined by LPA_1_-, LPA_2_- or LPA_3_-transfected CHO cells [15,19]. LPA_4–6_ receptors have been classified into the non-Edg family of LPA receptors and are structurally distant from the Edg family of LPA receptors [20]. The IC_50_ of LA-01 for LPA_4–6_ was not determined. Incubation with LA-01 did not affect viability of the FLSs (data not shown).

### Enzyme-linked immunosorbent assay

RA FLSs were cultured overnight in 96-well plates (2 × 10^4^ cells/well), then incubated with LA-01 (0, 1 or 10 nM) or Ki16425 (2 nM) (Cayman Chemical) 30 minutes before stimulation with LPA (10 μM) in FCS-free DMEM at 37°C for 24 hours. Protein levels of IL-6, chemokine (C-C motif) ligand 2 (CCL2), VEGF, matrix metalloproteinase 3 (MMP-3) and chemokine (C-X-C motif) ligand 12(CXCL12) in the culture supernatant were assessed by using ELISA kits (R&D Systems, Minneapolis, MN, USA) according to the instructions supplied by the manufacturer.

### Pseudoemperipolesis

FLSs were seeded onto 96-well plates (2 × 10^4^ cells/well) and cultured for 48 hours. CD4- and CD8-positive (CD4^+^ and CD8^+^, respectively) T cells and CD19^+^ B cells were purified from human peripheral blood of healthy volunteers by using MACS microbeads (>95% purity; Miltenyi Biotec, Auburn, CA, USA) and added to the FLS-cultured wells (1 × 10^5^ cells/well). The cells were treated with LA-01 (0, 1 or 10 nM) for 30 minutes, followed by stimulation with LPA (10 μM) in FCS-free DMEM. After 12 hours, the wells were washed three times with medium. Pseudoemperipolesis was assessed by counting the number of cells beneath FLSs in three independent fields under a microscope.

### Scrape motility assay

RA FLSs were plated at a density of 1 × 10^5^ cells/ml in 12-well plates in DMEM with 10% FCS. After overnight incubation, FLSs was washed twice with FCS-free medium. The tip of a plastic pipette was drawn across the center of the well to produce a scraped area. Culture wells were washed twice with PBS, and free cells were removed. After pretreatment with LA-01 (0, 1 or 10 nM) for 30 minutes, cells were incubated with LPA (10 μM) in FCS-free DMEM. A cell-free area was measured by using ImageJ software (National Institutes of Health, Bethesda, MD, USA) at 0 and 48 hours, and the ratio was then calculated (cell-free area at 48 hours per cell-free area at 0 hours).

### Expression of vascular cell adhesion molecule and intercellular adhesion molecule on RA fibroblast-like synoviocytes

FLSs were stimulated with LPA (10 μM) 30 minutes after adding LA-01 (0, 1 or 10 nM) in FCS-free DMEM at 37°C for 12 hours. Cells were stained with allophycocyanin-conjugated mAb against vascular cell adhesion molecule (anti-VCAM, clone STA; BioLegend) or phycoerythrin-conjugated mAb against intracellular adhesion molecule (anti-ICAM, clone HA58; eBioscience, San Diego, CA, USA). Allophycocyanin- or phycoerythrin-conjugated mouse IgG1 (BioLegend) was used as an isotype control. Cells were then analyzed by flow cytometry (Accuri C6 Flow Cytometer; BD Biosciences).

### Statistical analysis

Data are expressed as mean ± standard error of the mean (SEM). The comparison of the data from the two groups was conducted by using Student’s *t*-test. *P*-values less than 0.05 were considered significant.

## Results

### Expression of lysophosphatidic acid receptors in RA fibroblast-like synoviocytes

**Figure 1:**
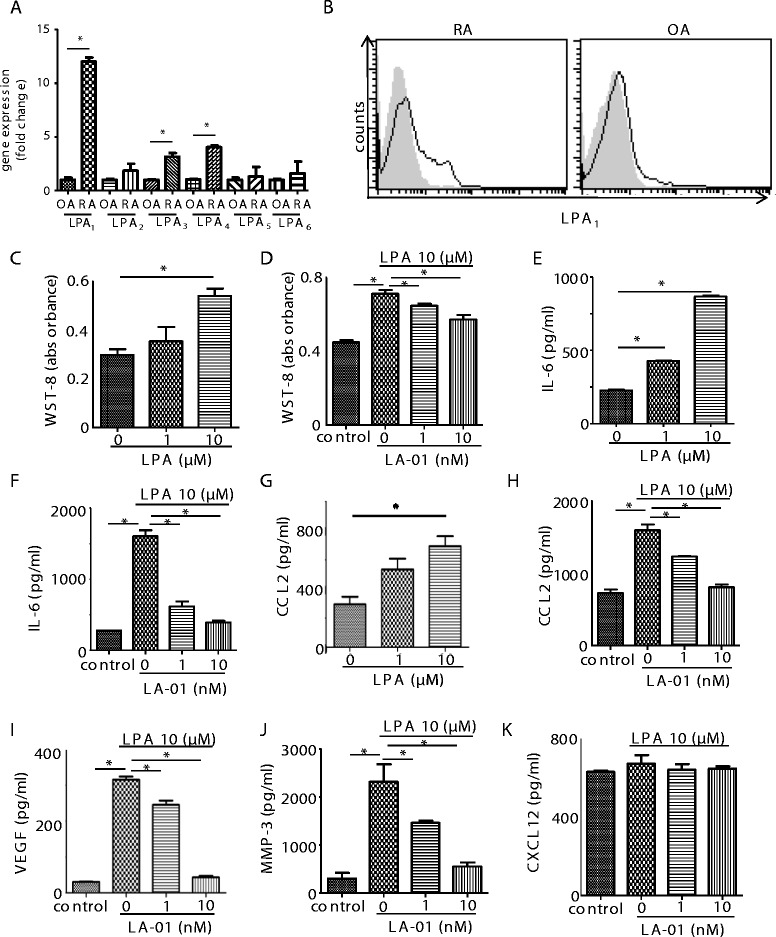
**Expression of lysophosphatidic acid receptors and the effect of lysophosphatidic acid receptor 1 on proliferation and production of inflammatory mediators in rheumatoid arthritis fibroblast-like synoviocytes.** The expression levels of lysophosphatidic acid receptor 1 through 6 (LPA_1–6_) mRNA in fibroblast-like synoviocytes (FLSs) derived from the rheumatoid arthritis (RA) synovium (n = 10) were compared to those in FLSs from osteoarthritis (OA) synovium (n = 5) by real-time RT-PCR **(A)**. Data were derived from samples from multiple individuals. Data are presented as the mean ± SEM. *P < 0.05 for RA vs OA. Cell surface expression of LPA_1_ on RA (n = 5) and OA (n = 3) FLSs was analyzed by flow cytometry **(B)**. Filled histogram (gray): isotype control; open histogram (black line): LPA_1_. Representative histograms are shown. RA FLSs were cultured with lysophosphatidic acid (LPA) for 72 hours **(C)**. FLSs were preincubated with an LPA1 inhibitor, LA-01, for 30 minutes,then stimulated with 10 μM LPA for 72 hours **(D)**. Control: no stimulation with LPA. Cell proliferation was measured by using a cell counting kit **(C)** and **(D)**. RA FLSs were cultured with LPA for 24 hours. Concentrations of interleukin 6 (IL-6) and chemokine (C-C motif) ligand 2 (CCL2) in the culture supernatant were measured by enzyme-linked immunosorbent assay (ELISA) **(E)** and **(G)**. FLSs were preincubated with LA-01 for 30 minutes, then stimulated with 10 μM LPA for 24 hours. Concentrations of IL-6, CCL2, vascular endothelial growth factor (VEGF), matrixmetalloproteinase (MMP-3) and CXCL12 in the culture supernatant were measured by ELISA **(F)**, and (H) through **(K)**. Control: no stimulation with LPA. Data are presented as the means (±SEM) of one of three independent experiments analyzed in triplicate. *P < 0.05 vs control or LA-01 0 nM **(C)** through **(K)**.

The expression of LPA_1–6_ mRNA in FLSs from RA and OA patients was analyzed by quantitative real-time RT-PCR. The expression of LPA_1_ mRNA in RA FLSs was significantly higher than that in OA FLSs (Figure 1A). The expression of LPA_3_ and LPA_4_ was also significantly higher in RA FLSs than that in OA FLSs, although the ratios of LPA_3_ and LPA_4_ expression in RA FLSs to OA FLSs were smaller than those of LPA_1_ expression. Cell surface LPA_1_ expression was analyzed by flow cytometry. RA FLSs were expressed LPA_1_ on the cell surface, and the expression level was substantially higher than that of OA FLSs (Figure 1B).

### Lysophosphatidic acid receptor 1 inhibitor suppressed lysophosphatidic acid–induced proliferation and cytokine production in RA fibroblast-like synoviocytes

We analyzed the effects of LPA on the proliferation and production of inflammatory mediators by RA FLSs. Stimulation with LPA dose-dependently induced the proliferation of FLSs (Figure 1C). LPA stimulation also induced the production of IL-6 and CCL2 from FLSs in a dose-dependent manner (Figures 1E and 1G), which supports a previous report that LPA upregulated IL-6 mRNA expression by RA FLSs [18]. Stimulation with LPA also induced the production of VEGF and MMP-3 by RA FLSs *in vitro* (Figures 1I and 1J).

Next, we analyzed the effect of an LPA_1_ inhibitor on LPA stimulation for RA FLSs. Enhanced cell proliferation by 10 μM LPA was significantly suppressed by LA-01, the LPA_1_-selective antagonist (Figure 1D). The treatment with LA-01 significantly reduced the production of IL-6, CCL2, VEGF and MMP-3 by LPA-stimulated RA FLSs (Figures 1F and 1H through 1J). In contrast, the production of CXCL12 by RA FLSs was not altered by stimulation with LPA (Figure 1K). We used Ki16425, another LPA_1_ antagonist, to confirm the effects of LPA_1_ inhibition on IL-6 production from LPA-stimulated RA FLSs. Incubation with Ki16425 suppressed IL-6 production from LPA-stimulated RA FLSs as well as LA-01 (IL-6 concentrations: vehicle = 299.413 ± 28.084 pg/ml; Ki16425 = 116.785 ± 11.162 pg/ml (*P* < 0.05 vs vehicle); LA-01 = 145.715 ± 15.921 pg/ml (*P* < 0.05 vs vehicle)). These results suggest that LPA–LPA_1_ signaling plays important roles in proliferation and cytokine production of RA FLSs *in vitro*.

### LPA–LPA_1_ signaling promoted pseudoemperipolesis

**Figure 2:**
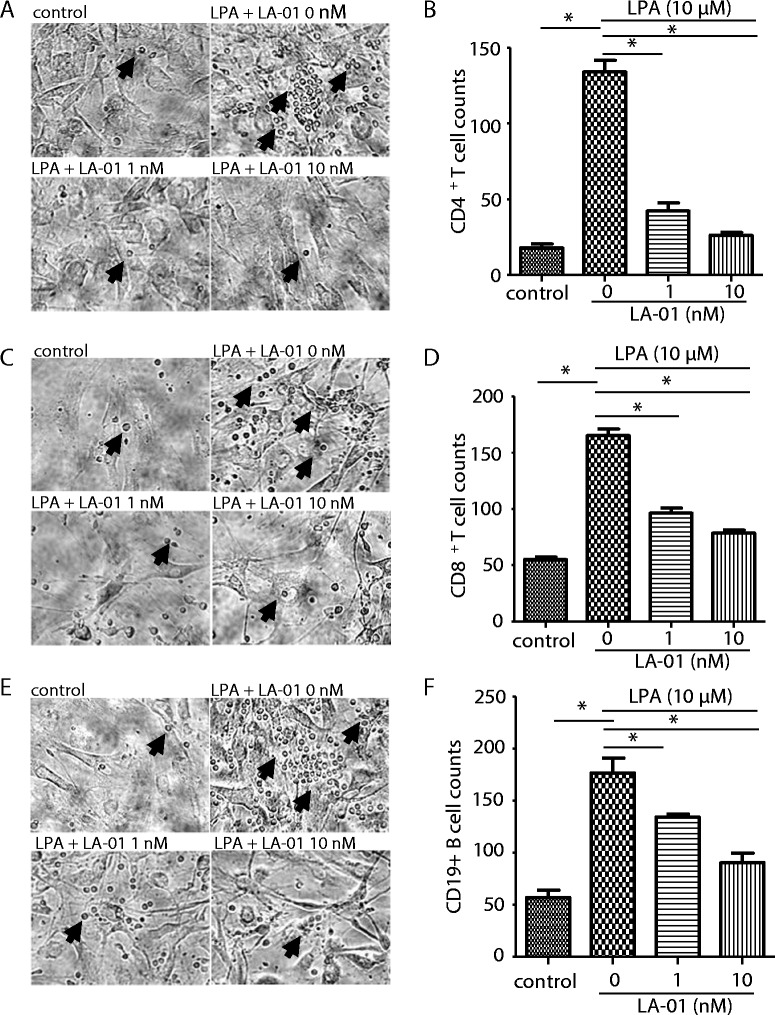
**Effect of lysophosphatidic acid receptor 1 on pseudoemperipolesis and migration of rheumatoid arthritis fibroblast-like synoviocytes.** After preincubation of cocultured rheumatoid arthritis (RA) fibroblast-like synoviocytes (FLSs) and CD4^+^ T cells **(A)** and **(B)** or CD8^+^ T cells **(C)** and **(D)** or CD19^+^ B cells **(E)** and **(F)** with a lysophosphatidic acid (LPA) receptor 1 inhibitor (LA-01; 0, 1 or 10 nM) for 30 minutes, the cells were stimulated with 10 μM LPA for 12 hours. Control: no stimulation with LPA. After the cells were washed, the number of lymphocytes beneath FLSs was counted. Representative photomicrographs of three independent experiments are shown **(A, C and E)**. Arrows indicate the lymphocytes beneath FLSs. Original magnification, ×200. Data on the number of lymphocytes beneath FLSs are presented as one of three independent experiments analyzed in triplicate **(B, D, and F)**. Data are presented as the mean ± SEM. *P < 0.05 vs control or LA-01 0 nM **(B, D, F)**.

RA FLSs have been shown to promote the spontaneous migration of leukocytes beneath them, a process termed *pseudoemperipolesis* [21]. We examined the effect of LPA on pseudoemperipolesis. Stimulation with 10 μM LPA significantly increased the number of CD4^+^ and CD8^+^ T cells, as well as CD19^+^ B cells, beneath RA FLSs (Figures 2A to 2F). Moreover, incubation with LA-01 suppressed the LPA-enhanced pseudoemperipolesis of CD4^+^ and CD8^+^ T and CD19^+^ B cells (Figures 2A through 2F), suggesting that interaction of LPA and LPA_1_ promotes pseudoemperipolesis of leukocytes.

### LPA–LPA_1_ signaling promoted cell motility of RA fibroblast-like synoviocytes

**Figure 3:**
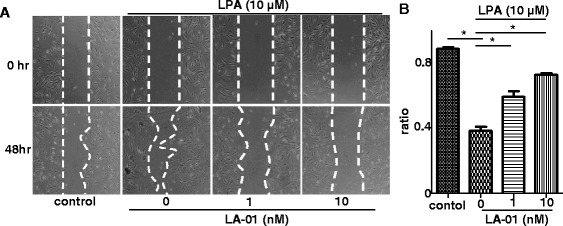
**The effect of lysophosphatidic acid receptor 1 on the migration of rheumatoid arthritis fibroblast-like synoviocytes.** A scraped cell-free area was created on cultured RA FLSs. After preincubation with a lysophosphatidic acid (LPA) receptor 1 inhibitor (LA-01; 0, 1 or 10 nM) for 30 minutes, cells were stimulated with 10 μM LPA for 48 hours. Control: no stimulation with LPA. **(A)** Representative photomicrographs of three independent experiments are shown. Original magnification, ×40. **(B)** The cell-free area was assessed, and a ratio (cell-free area in 48 hours to cell-free area in 0 hours) was defined. Data are presented as the means (±SEM) of one of three independent experiments analyzed in triplicate. *P < 0.05 vs control or LA-01 0 nM. Upper dashed line indicates the cells are stimulated with LPA 10 uM, and lower dashed line indicates LA-01 is added with indicated concentration.

We also analyzed the effect of LPA_1_ on RA FLS migration by scrape motility assay. Incubation with 10 μM LPA significantly decreased the cell-free area, indicating that LPA induced cell migration *in vitro* (Figures 3A and 3B), as reported previously [22]. In addition, LA-01 significantly increased the cell-free area of RA FLSs (Figures 3A and 3B), suggesting that LPA–LPA_1_ signaling also contributes to the promotion of RA FLS motility.

### LPA–LPA_1_ signaling induced adhesion molecule expression on RA fibroblast-like synoviocytes

**Figure 4:**
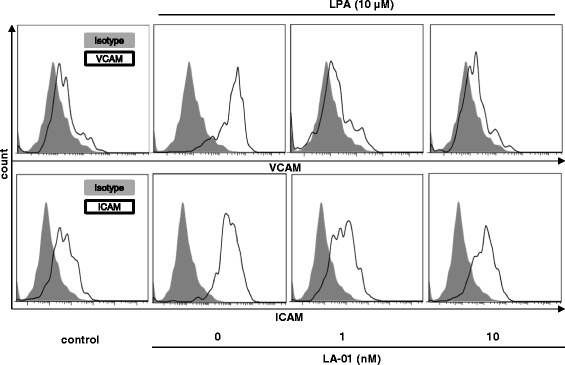
**The effect of lysophosphatidic acid receptor 1 on the expression of adhesion molecules on fibroblast-like synoviocytes.** Rheumatoid arthritis (RA) fibroblast-like synoviocytes (FLSs) were pretreated with a lysophosphatidic acid (LPA) receptor 1 inhibitor (LA-01; 0, 1 or 10 nM) for 30 minutes, then the cells were stimulated with 10 μM LPA for 12 hours. Cells were stained with allophycocyanin-conjugated monoclonal antibody (mAb) against vascular cell adhesion molecule (anti-VCAM) or phycoerythrin-conjugated mAb against intercellular adhesion molecule (anti-ICAM). Allophycocyanin- or phycoerythrin-conjugated mouse immunoglobulin G1 (IgG1) was used as a control. The expression of VCAM and ICAM on FLSs was analyzed by flow cytometry. Filled histogram (gray): isotype control; open histogram (black line): VCAM or ICAM.

It has been reported that signaling from VCAM and ICAM in RA FLSs supports pseudoemperipolesis [21]. Therefore, we next analyzed the expression of VCAM and ICAM on RA FLSs by flow cytometry. We found that stimulation with 10 μM LPA induced the expression of VCAM and ICAM on RA FLSs (Figure 4). Moreover, LA-01 decreased the expression of VCAM and ICAM induced by LPA on RA FLSs (Figure 4). However, the expression of E-selectin on RA FLSs was not altered by LPA simulation (data not shown).

## Discussion

In this study, we found that LPA_1_ was highly expressed in RA FLSs. LPA stimulated RA FLSs to enhance proliferation, production of inflammatory mediators, pseudoemperipolesis, migration and the expression of adhesion molecules, which are attributable to signaling through LPA_1_.

RA FLSs express inflammatory cytokines, chemokines and matrix-degrading enzymes, which contribute to the pathogenesis of RA. LPA has been reported to induce IL-6 mRNA expression on RA FLSs, as well as cell motility [13]. However, the corresponding LPA receptor on RA FLSs has not been identified. We show that LPA augmented IL-6, CCL2, VEGF and MMP-3 production by RA FLSs. Moreover, the LPA-induced production of the inflammatory mediators was inhibited by a LPA_1_-selective inhibitor. Therefore, the LPA–LPA_1_ cascade plays an important role in cytokine, chemokine and matrix-degrading enzyme production by RA FLSs. Although IC_50_ of LA-01 was 86 nM, which was determined by using LPA_1_-transfected CHO cells, 10 nM LA-01 significantly inhibited stimulation of LPA in RA FLSs. The IC_50_ may be dependent on cell type or on the expression level of LPA_1_.

Pseudoemperipolesis contributes to the chronic inflammation induced by lymphocyte recruitment in the inflamed joints and protects lymphocytes from apoptosis [21,23,24]. We show that LPA enhanced the pseudoemperipolesis of T and B cells, which is also attributable to LPA_1_. It has been reported that stimulation with CXCL12 and signaling from VCAM and ICAM in RA FLSs support pseudoemperipolesis [21]. Our results indicate that LPA upregulated the expression of VCAM and ICAM on RA FLSs, which was blocked by the LPA_1_ antagonist. Thus, LPA may enhance pseudoemperipolesis via the upregulation of VCAM and ICAM expression on RA FLSs through LPA_1_. Interestingly, CXCL12 production by RA FLSs was not altered by LPA simulation. Stimulation of lymphocytes by LPA via LPA_1_ may also contribute to the enhanced pseudoemperipolesis. In this regard, it has been reported that LPA induced chemokinesis in T cells [25] and lymphocyte transmigration through high endothelial venules [26,27]. Further studies are needed to clarify the effects of LPA–LPA_1_ signaling for the lymphocytes on pseudoemperipolesis.

The hyperplastic rheumatoid pannus is characterized by an overabundance of FLSs [2]. This cellular excess stems largely from an imbalance between the proliferation and apoptosis of FLSs [2]. The migration of RA FLSs may also contribute to pannus formation [2]. Our results show that LPA induced the proliferation and migration of FLSs, which was inhibited by the LPA_1_ antagonist. Moreover, in a recent study, researchers reported that LPA suppressed tumor necrosis factor–induced apoptosis on RA FLSs via LPA_1_ [28]. Therefore, it is suggested that the LPA–LPA_1_ signaling also contributed to the cellular excess and migration of FLSs in the RA synovium.

In this study, we show that there are important roles of LPA–LPA_1_ signaling on RA FLS stimulation. However, the effects of LPA signals via LPA_2–6_ remain unclear, although RA FLSs also expressed LPA_2–6_. Further studies are warranted to elucidate the roles of LPA_2–6_ in LPA stimulation of FLSs by using each of the LPA receptor–specific antagonists or FLSs from each LPA receptor–deficient mouse.

It was shown that conditional genetic ablation of ATX, which generates LPA via hydrolysis of lysophosphatidylcholine, in mesenchymal cells resulted in disease attenuation in animal models of arthritis [12]. We have also found that LPA_1_ is essential for the development of arthritis in collagen-induced arthritis [15]. The ATX–LPA–LPA_1_ axis may play an important role in the development of arthritis.

## Conclusion

Our study suggests that LPA–LPA_1_ signaling in FLSs may contribute to the pathogenesis of RA by inducing proliferation, production of inflammatory mediators, pseudoemperipolesis and migration on RA FLSs. Thus, LPA_1_ could be a promising therapeutic target for RA.

## Abbreviations

ATX: Autotaxin
CCL2: Chemokine (C-C motif) ligand 2
CXCL12: Chemokine (C-X-C motif) ligand 12
DMEM: Dulbecco’s modified Eagle’s medium
Edg: Endothelial cell differentiation gene
ELISA: Enzyme-linked immunosorbent assay
FCS: Fetal calf serum
FLS: Fibroblast-like synoviocyte
ICAM: Intercellular adhesion molecule
IL: Interleukin
LPA: Lysophosphatidic acid
mAb: Monoclonal antibody
MMP-3: Metalloproteinase 3
OA: Osteoarthritis
RA: Rheumatoid arthritis
SEM: Standard error of the mean
VCAM: Vascular cell adhesion molecule
VEGF: Vascular endothelial growth factor
